# Reproductive Strategies of the Swelled Vent Frog (*Nanorana quadranus*): Testicular Size, Sperm Traits, and Fecundity Responses to Geographical Gradients

**DOI:** 10.3390/biology14091224

**Published:** 2025-09-09

**Authors:** Lulu Lyu, Shuang Huang, Miao He, Yan Huang

**Affiliations:** 1 Key Laboratory of Southwest China Wildlife Resources Conservation (Ministry of Education), China West Normal University, Nanchong 637009, China; 15881710835@163.com (L.L.); shuang_huang1230@163.com (S.H.); 17609268594@163.com (M.H.); 2 Key Laboratory of Artificial Propagation and Utilization in Anurans of Nanchong City, China West Normal University, Nanchong 637009, China

**Keywords:** *Nanorana quadranus*, reproductive strategy, environmental variation, testicular investment, sperm morphology, female fecundity

## Abstract

Reproductive strategies represent a fundamental aspect of life-history evolution and are shaped by environmental heterogeneity across geographic gradients. This study investigated geographic variation in reproductive traits of the swelled vent frog (*Nanorana quadranus*), a stream-breeding species in China’s Qinling–Daba Mountains. Male reproductive traits were assessed across 10 populations, including testicular asymmetry, relative testis size, sperm morphology, and sperm count. Female reproductive traits were examined in 12 populations, focusing on body mass and absolute fecundity. Results indicated no significant difference in bilateral testicular asymmetry (*p* > 0.05). Both relative testis size and sperm count increased with latitude. Sperm length correlated positively with testis size. Conversely, female body mass and age increased with altitude, while absolute fecundity was positively correlated with body mass. Environmental analysis revealed that sperm length exhibited significant positive correlations with aspect and seasonal evapotranspiration anomaly (SEA). Relative testis size was regulated by mean diurnal temperature range (Bio2) and precipitation of the wettest month (Bio13). Furthermore, female absolute fecundity correlated with the minimum temperature of the coldest month (Bio6). These findings demonstrate that reproductive strategies in *N. quadranus* adapt to geographic and environmental gradients, reflecting adaptive plasticity to local ecological pressures. This study advances understanding of amphibian reproductive adaptation and highlights the importance of incorporating environmental factors in life-history research.

## Simple Summary

Geographic variation in reproductive strategies reflects adaptations to environmental stress beyond body size and physiology. This study examines such variation in the swelled vent frog (*Nanorana quadranus*). Results showed that both relative testis size and sperm count increased with latitude. Sperm length also correlated positively with testis size. Female body mass and age increased with altitude, while absolute fecundity was positively correlated with body mass. Environmental analyses revealed that sperm length exhibited significant positive correlations with aspect and seasonal evapotranspiration anomaly (SEA). Relative testis size was regulated by mean diurnal temperature range (Bio2) and precipitation of the wettest month (Bio13). Female absolute fecundity correlated positively with the minimum temperature of the coldest month (Bio6).

## 1. Introduction

Reproductive strategies encompass a suite of behavioral, morphological, and physiological adaptations that enable species to optimize resource allocation, enhance mating and fertilization success, and improve offspring survival under specific environmental conditions [1]. As a cornerstone of ecological and evolutionary biology, the study of reproductive strategies provides critical insight into how species adapt to heterogeneous environments to ensure reproductive success and lineage continuity [2]. Since Darwin, sexually dimorphic traits that enhance reproductive fitness have remained central to theories of sexual selection and life-history evolution [3]. Accumulating empirical evidence suggests that ecological gradients—such as latitude, altitude, ambient temperature, resource availability, predation pressure, and interspecific competition—play a fundamental role in shaping reproductive traits across populations. These gradients often drive trade-offs in energy allocation between somatic growth and reproduction, as well as between offspring number and size, reflecting divergent adaptive strategies [4,5,6,7,8]. Recent research across diverse taxa, including birds, mammals, fish, amphibians, and insects, has revealed substantial geographic variation in reproductive traits and strategies [9,10,11,12,13,14]. These studies underscore the importance of environmental conditions in driving population-level divergence in reproductive allocation and life-history patterns [15].

Currently, research on geographic variation in reproductive strategies primarily focuses on correlations among male morphological traits (e.g., testis size, sperm length) and female reproductive trade-offs between clutch mass and clutch size [16,17,18]. Anuran populations frequently exhibit male-biased sex ratios, which intensify intrasexual competition [19,20]. This competition is expressed mainly through two mechanisms: (i) pre-copulatory contests, such as male–male competition in monogamous species, and (ii) post-copulatory sperm competition, mainly observed in polygamous mating systems [21,22,23]. Typically, species evolve to favor one form of competition over the other. Under conditions of strong male–male competition, selection favors pre-mating investment in traits such as enhanced musculature or weaponry, whereas in polygamous systems, males tend to invest in sperm competition by developing larger testes and producing greater sperm counts [24,25]. In males, testis size and sperm length are key indicators of reproductive strategy [26,27], with variation influenced by reproductive mode, male–male competition intensity, and geographic gradients. Sperm competition theory posits that in polygynous mating systems, males enhance reproductive success by increasing relative testis size (total left and right testes mass/snout-vent length (SVL)) or by balancing sperm length and count [28]. In amphibians, high-altitude environments are typically characterized by shorter breeding seasons and higher reproductive synchrony, which intensify male–male competition and sperm competition [20]. These factors are theoretically predicted to promote the evolution of larger testes and elongated sperm in males inhabiting high elevations [29]. Consistent with this prediction, species such as *Duttaphrynus melanostictus* demonstrate altitudinal increases in testis size [30]. However, contrasting patterns are observed in *Crinia georgiana* and *Rana temporaria*, where testis size decreases with increasing altitude or latitude [31,32]. Furthermore, research on *Dianrana pleuraden* reveals that testis size does not increase with altitude or male operational sex ratio (OSR) [23]. These contradictions may arise from differential energy allocation within male competitive strategies: under intense competition for mates, males may adopt distinct energy allocation patterns between male–male competition and sperm competition [33].

Female reproductive strategies primarily involve the energy allocation trade-offs between clutch mass and clutch size, influenced by factors such as body size, reproductive mode, spawning frequency, and environmental conditions [7,34,35]. In amphibians, the quantity–quality trade-off is evident, with optimal combinations of clutch mass and egg number reflecting life-history adaptations to environmental gradients [36]. Life-history theory posits that in cold environments (e.g., high altitudes/latitudes), females invest more in ovarian development to produce fewer but larger eggs, enhancing offspring survival by allocating more energy reserves per progeny and compensating for harsh developmental conditions [37,38]. In contrast, smaller ovaries are usually associated with smaller eggs and larger clutch sizes, which enhances female reproductive output [39,40]. Studies have shown that species such as the *Bufo andrewsi*, *Rhacophorus omeimontis*, and *Rana sylvatica* exhibit a characteristic of fewer eggs and relatively larger ovaries in high-altitude areas [35,41,42]. Additionally, *Geocrinia victoriana* adjusts the combination of clutch size and clutch mass during different reproductive stages in unstable habitats to compensate for the resource allocation of a single reproductive event [43]. Amphibians with slower life histories typically produce both larger eggs and larger clutch sizes compared to faster-lived species [44]. However, the adaptive value of different combinations of clutch mass and clutch size varies with environmental changes [45,46,47].

Amphibians exhibit high vulnerability to climate change due to their permeable skin, biphasic aquatic-to-terrestrial life cycle, and dependence on environmental thermoregulation [48,49,50,51,52]. Their limited dispersal capacity and diverse reproductive strategies further establish them as exemplary models for investigating reproductive adaptations. Among environmental variables, temperature plays a pivotal role in shaping amphibian abundance and distribution by regulating key physiological processes such as breeding phenology, growth rates, and energy metabolism [53]. Likewise, reduced precipitation, by disrupting the water-dependent reproductive ecology of anurans, significantly reduces body size, thereby directly compromising reproductive success and increasing the likelihood of reproductive failure [50,54]. Empirical evidence demonstrates that under drought-induced resource constraints, *Pseudophryne guentheri* develops significantly reduced testis size and sperm length [26,55], exemplifying direct climatic shaping of reproductive traits. Climate change broadly modulates amphibian behavioral, morphological, and physiological parameters through thermal influences on phenology and precipitation-mediated regulation of population dynamics [56,57]. Given intense selective pressures on climate-associated traits, systematic examination of adaptive reproductive strategies in amphibians is essential to understand the evolutionary mechanisms that underlie adaptation to ongoing environmental change.

The swelled vent frog *(Nanorana quadranus)*, a stream-breeding species endemic to China’s Qinling–Daba Mountains (altitude: 335–1830 m), is the most widespread, altitudinally diverse, and abundant member of its genus [58]. It was last assessed by the IUCN Red List in 2019 and classified as Near Threatened (NT) under criteria A2acd, reflecting an inferred population decline driven by habitat loss, environmental degradation, and overexploitation [59]. Its complex metamorphosis (gill-breathing larvae to lung-breathing adults) renders it particularly sensitive to environmental disturbances. Its unique amplexus-free reproductive mode [60] makes it a key target for investigating geographic variation in reproductive strategies [61]. Although studies have characterized its anatomy [33,62] and phylogenetic relationships [58,63,64], information regarding geographic variation in male reproductive investment (e.g., testicular allometry, sperm morphology) and female fecundity remains lacking in systematic analysis.

Given the high ecological sensitivity of amphibians and the current threat status of over 40% of species [65,66,67], deciphering the reproductive strategies of *N. quadranus* is essential for elucidating mechanisms of adaptive evolution and supporting long-term species conservation. This study aimed to examine how reproductive traits in *N. quadranus* vary geographically and respond to environmental gradients across altitudinally diverse populations. Specifically, male reproductive characteristics, including absolute and relative testis size and sperm traits, and female reproductive traits, such as clutch mass and clutch size, were quantitatively assessed in relation to climatic and topographic variables. By integrating morphological and ecological data, this study provides novel insights into the environmental drivers of reproductive trait divergence and contributes to a broader understanding of amphibian life-history adaptation in heterogeneous habitats.

## 2. Materials and Methods

### 2.1. Fieldwork and Sampling

**Figure 1:**
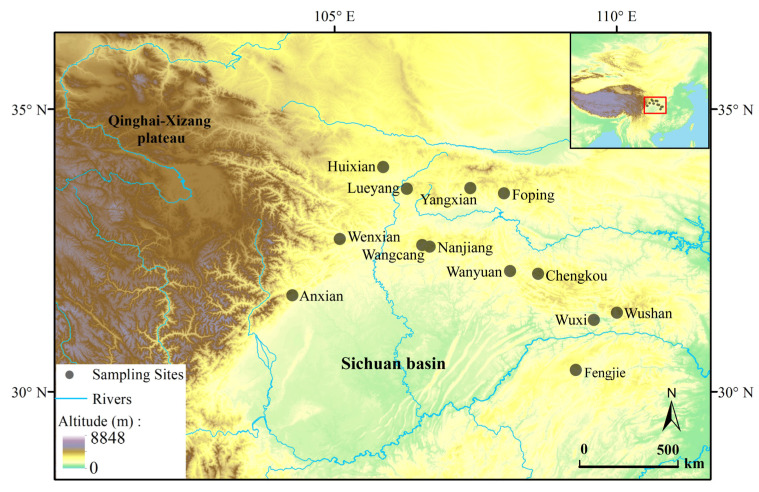
Geographic distributions of 13 *Nanorana quadranus* population samples; sampling site coordinates are listed in Supplementary Table S1.

A total of 266 *N. quadranus* individuals, including 134 males and 132 females, were collected from 13 sampling sites during the breeding seasons of 2020 and 2021. The distribution of individuals across the sampling sites is detailed in Table S1, which includes the number of males and females sampled at each location, while their geographic coordinates are visually presented in Figure 1. The main locations were situated in the mountainous areas of Sichuan, Chongqing, Shaanxi and Gansu, with an altitude range of 586–1702 m. In addition, the latitude, longitude and altitude of each location were recorded using a GPS positioning system (Two Steps Outdoor Assistant, Shenzhen 2bulu Information Technology Co. Ltd., Shenzhen, China). The samples were transported back to the laboratory, where all 266 individuals were first anesthetized with benzocaine solution before being processed [62]. Subsequently, SVL (mm) was measured with electronic digital calipers, accurate to 0.02 mm [68]. Body mass (g) was determined using an electronic balance, accurate to 0.0001 g. To ensure consistency and accuracy, all measurements were taken by the same person. The third toe from the left foot of each frog was removed, and the phalangeal bone was placed in a 10% formalin solution for age determination (see below). The specimens were preserved in 4% buffered formalin in a phosphate buffer [69]. All procedures involving animals were approved by the Animal Ethics Committee of China West Normal University.

### 2.2. Age Determination

Skeletochronology is considered the most reliable method for age estimation in wild amphibians and is based on counting lines of arrested growth (LAGs) in long bones [69,70,71,72]. Paraffin sectioning and Harris’ hematoxylin staining were used to prepare histological sections (see [73] for details). Toes fixed in 10% formaldehyde for more than 10 days were washed in water for 12 h, decalcified in 5% nitric acid for 14 h, rinsed in running tap water overnight, and stained with Ehrlich’s hematoxylin for 3.5 h. The stained bones were dehydrated and embedded in paraffin blocks. The cross-section of the finger bone with the smallest medullary cavity and the thickest cortical bone (about 13 µm thick) was selected and fixed on a slide. LAG counts were recorded from mid-diaphyseal sections under light microscopy, with validation from four marked–recaptured individuals confirming that LAGs reflect actual age [74]. Endosteal resorption of LAGs was assessed by comparing the minimum cross-sectional diameter of 1-year-old *N. quadranus* with the diameter of the resorption line in adult individuals [75]. Double lines and false lines were readily distinguishable from true LAGs, minimizing age estimation errors [76,77]. A total of 266 specimens (134 males and 132 females) displayed distinct LAGs in their bone sections.

### 2.3. Testicular Morphology and Sperm Analysis in Males

A total of 133 male subjects had their right and left testes surgically excised from the abdominal cavity (Table S3). The number of male individuals included in this study was primarily determined by field collection success. During the procedure, the testes were accurately weighed using an electronic balance with an accuracy of ±0.1 mg, transferred to pre-weighed sterilized Petri dishes (M1), homogenized, and mixed with distilled water to form a sperm suspension (M2). Suspension mass (X) was calculated as M2 − M1. The number of spermatozoa in the five middle squares was counted using a hemocytometer (Shanghai Seeking Precision Biochemical Reagent & Instrument Co., Ltd. Shanghai, China). The number of spermatozoa in 1 cm^3^ of the suspension was estimated to be 5a × 10^4^. The total number of spermatozoa, combined with the mass of the suspension, was calculated to be 5a × 10^4^ × X [78].

A subset of 56 samples was selected for sperm characterization (Table S5). Sperm suspensions were uniformly smeared onto glass slides, air-dried, and fixed in 95% ethanol for 5–10 min. After drying, the slides were stained with Ehrlich’s hematoxylin for 25 min, washed with distilled water, differentiated in tap water for 20–30 min, washed with distilled water, stained with 1% eosin for 15 min, washed with distilled water, air-dried, and photographed under a 40× Motic DM-BA300 digital microscope (Motic (Xiamen) Electric Group Co., Ltd. Xiamen, China). Head and tail lengths of twenty intact spermatozoa per sample were measured in triplicate using Motic Images Advanced 3.2 software, with mean values recorded to a resolution of 0.01 μm [78,79,80].

### 2.4. Fecundity Estimation in Females

A total of 132 female individuals from 12 populations were dissected via midventral incision of the body (Table S6). The difference in the number of male and female samples, resulting from uncontrollable factors in field sampling, primarily reflects the natural population sex ratio characteristics of *N. quadranus*, with no sex-biased selection applied during collection. The skin and muscle were removed, and then the ovaries were extracted and weighed using an electronic balance (accurate to ±0.0001 g). One-third of each ovary was weighed, and the number of eggs was counted using a mechanical counter. Clutch size was quantified as the number of mature ova per individual. In the present study, a mature ovum was defined as an independent, relatively uniform-sized sphere with a distinct boundary within the ovary [81]. This clutch size was extrapolated to estimate total fecundity using the formula [82] Total clutch size = (Clutch size/(1/3 clutch mass)) × Total clutch mass.

### 2.5. Environment Variables

To explore the influence of environmental factors on the reproductive strategies of *N. quadranus*, this study classified five environmental categories: temperature, precipitation, energy availability, primary productivity, and topography [83,84]. The latter critically shapes reproductive success by creating microhabitat heterogeneity, regulating hydrological conditions, and facilitating dispersal corridors for breeding site selection [85]. To further analyze these relationships, 25 environmental variables were selected for ecological modeling and analysis [86,87]. Climate data (19 variables) were sourced from https://worldclim.org (accessed on 20 July 2021) [88], including temperature-related indices (Bio1–Bio11) and precipitation-related indices (Bio12–Bio19). Energy utilization variables—Potential Evapotranspiration (PET), Actual Evapotranspiration (AET), and Seasonal ET Anomaly (SEA)—were obtained from https://www.usgs.gov/products/data (accessed on 20 July 2021). Normalized Difference Vegetation Index (NDVI) data, representing productivity, were acquired from https://www.resdc.cn/ (accessed on 20 July 2021) [89]. Digital Elevation Model (DEM) data were retrieved from http://www.gscloud.cn/ (accessed on 20 July 2021), from which topographic derivatives (slope, aspect) were derived (Table S2). Environmental variables for all sampling sites were extracted using ArcGIS 9.2 (ESRI, Redlands, CA, USA) [68].

### 2.6. Statistical Analyses

All analyses were conducted using IBM SPSS Statistics 22.0 (Statistical Product and Service Solutions Company, Chicago, IL, USA). Parametric or non-parametric tests were applied as appropriate. Statistical tests were two-tailed, with significance levels set at *p* < 0.05 and high significance at *p* < 0.01. Data visualization was performed using the ggplot2 package in R software (version 4.3.3) [90].

To compare the differences between male and female *N. quadranus* in age distribution, body mass, SVL, and growth patterns, this study utilized boxplots, scatter plots, and statistical tests, including the Wilcoxon rank-sum test and *t*-tests. Additionally, gender-specific linear regression analysis was performed to examine differences in growth rates, revealing a trade-off between growth and reproductive investment. To analyze testicular symmetry (left–right) across 10 male populations, paired-sample *t*-tests were employed. Asymmetry was quantified using both absolute asymmetry (|Left–Right testis mass|) and relative asymmetry ([2 × |L − R|]/Total testis mass) following Møller and Swaddle [91]. General Linear Models (GLMs) were used to assess population differences in testicular asymmetry, with altitude as a fixed factor. Post hoc comparisons were made using Fisher’s Least Significant Difference (LSD) test. SVL, body mass, age, and testis size were included as covariates for adjustment. Spearman’s rank correlation was used to examine associations between relative/absolute asymmetry and body size or age. Relative testis size differences among populations were compared using One-Way Analysis of Variance (ANOVA), followed by LSD post hoc tests. The influence of SVL and age was controlled for using GLM. Regression and correlation analyses were employed to examine the relationship between testis size in *N. quadranus* and both altitude and latitude. Population differences in sperm size (head + tail length) were tested using the Kruskal–Wallis test. Associations between sperm size and environmental factors were analyzed using linear mixed models. GLMs were also used to test sperm size differences with altitude as a fixed factor, followed by LSD post hoc tests. To account for potential confounding effects, SVL, body mass, age, and testis size were included as co-variates in the GLMs. These models were implemented to test population-level differences in sperm count, with altitude treated as a fixed factor. Post hoc pairwise comparisons were conducted using an LSD test, while sperm size was additionally included as a covariate in these analyses. Additionally, Spearman’s rank correlation analysis was employed to assess associations between sperm size/count and SVL, body mass, and age. Spearman’s rank correlation was used to assess correlations between sperm size, sperm count, and testis size within *N. quadranus*.

Differences in female body mass and SVL among populations were analyzed using one-way ANOVA. ANCOVA was used to control for age. A GLM was used to explore the interactive effect of age and altitude on body mass. Absolute reproductive investment was measured as total egg volume per individual. Population differences were assessed using the Kruskal–Wallis test. The influence of SVL, body mass, and age was controlled for using ANCOVA. Correlation and regression analyses were performed to investigate the relationships between SVL, body mass, age, and absolute reproductive investment.

To investigate associations between reproductive traits and environmental factors, 25 environmental variables were extracted using ArcGIS 9.2 from 10 male and 12 female populations. For male relative testis size and sperm size, simple linear regression was used to screen variables. Subsequently, based on the Akaike Information Criterion (AIC) [92], ordinary least squares (OLS) multiple regression models were constructed. For relative testis size, predictor variables included NDVI, Bio2 [93], Bio13, aspect, and SEA. For sperm size, predictors included NDVI, Bio2, Bio18, aspect, slope, and SEA. For female absolute reproductive investment, simple linear regression was first used to screen associations. An AIC-based OLS multiple regression model was then built using NDVI, Bio6, Bio18, aspect, AET [50], and SEA as predictors. All multivariate models treated the respective reproductive trait as the response variable and environmental variables as predictors.

## 3. Results

### 3.1. Sexual Dimorphism in Age Distribution, Body Size, and Growth Patterns of N. quadranus

**Figure 2:**
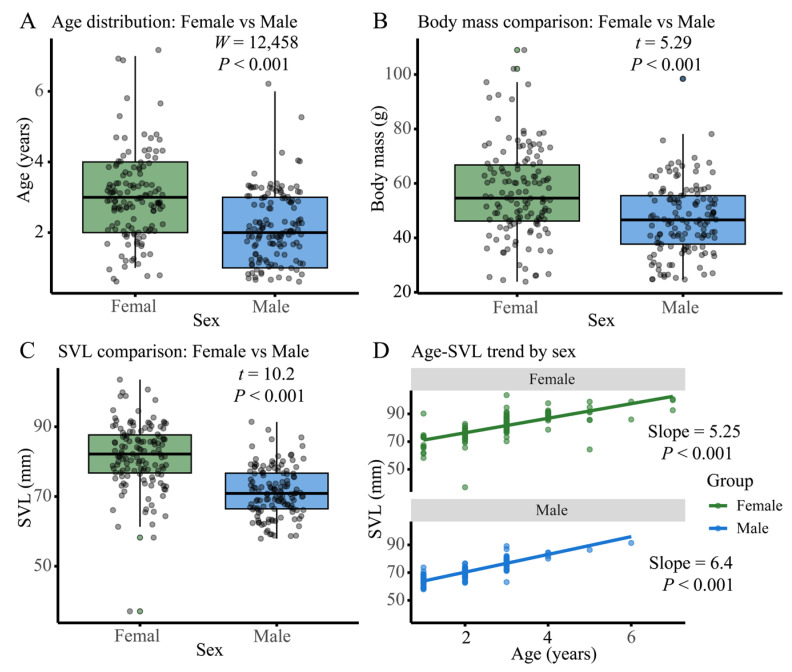
Sexual dimorphism and trait relationships in *N. quadranus*. Boxplots and regression analyses characterize sexual dimorphism in *N. quadranus*: (**A**) (Age Distribution): Females show broader age dispersion, with median age similar to males but more outliers. (**B**) (Body Mass): Females have higher median mass and greater variability, likely linked to reproductive energy demands. (**C**) (SVL): Females exhibit longer median SVL, aligning with fecundity-related body size investment. (**D**) (Age–SVL Trend): Females display steeper SVL growth with age, suggesting sex-specific growth trade-offs for reproduction. These traits indicate female-biased dimorphism in *N. quadranus*, influencing population dynamics (e.g., mating, resource use).

Analysis of sexual dimorphism characteristics (Figure 2) revealed significant differences between male and female *N. quadranus* in age distribution, body mass, SVL, and growth patterns. Boxplots with overlaid scatter points and statistical tests showed that although the age distributions of females and males overlapped (with similar medians), females exhibited greater age dispersion. The Wilcoxon rank-sum test confirmed a significant difference (*W* = 12,458, *p* < 0.001; Figure 2A). Males had a significantly lower median body mass with smaller dispersion (*t* = 5.29, *p* < 0.001; Figure 2B), while the high dispersion of female body mass might be related to reproductive energy allocation strategies. In terms of SVL, females had a significantly longer median SVL (*t* = 10.2, *p* < 0.001; Figure 2C), which is consistent with the hypothesis of fecundity-driven selection for larger body size. Further gender-specific linear regression analysis of body length against age showed that males had a steeper growth rate (b = 6.4, *p* < 0.001), while females had a relatively gentler growth rate (b = 5.25, *p* < 0.001) (Figure 2D). This differential growth pattern suggests a trade-off between growth and reproduction, which not only shapes the sexual dimorphism of *N. quadranus* but also influences population-level processes such as mating dynamics and resource allocation.

### 3.2. Geographical Variation in Testis Asymmetry and Relative Size

Paired-sample *t*-tests across *N. quadranus* populations revealed no bilateral asymmetry in testis size (all groups: *p* > 0.05; Table S3). GLMs indicated non-significant variation in absolute asymmetry (F_9, 123_ = 1.401, *p* = 0.195) or relative asymmetry (F_9, 123_ = 1.436, *p* = 0.180) among populations. Post hoc LSD tests revealed greater asymmetry in the 1518 m altitude population versus other altitudinal groups (*n* = 14; absolute asymmetry: *p* < 0.05; relative asymmetry: *p* < 0.05), while no significant interpopulation differences were found otherwise (*p* > 0.05). Spearman’s correlation analysis revealed no significant correlations between relative asymmetry and absolute asymmetry, SVL, age, or body mass (*p* > 0.05).

**Figure 3:**
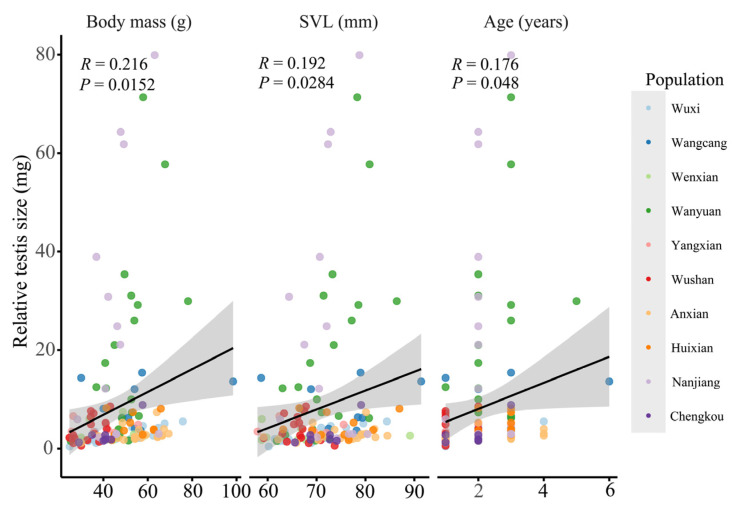
Relationships between relative testis size and body mass, SVL, and age among populations of *N. quadranus*. The scatter plots are divided into three columns to present the associations between relative testis size and body mass, SVL, and age, respectively. The 10 distinct populations are represented by scatter points of different colors (see the legend for population code correspondences; specific sample sizes are provided in Table S4). Black solid lines indicate significant linear regression results, and the gray shaded areas represent the 95% confidence intervals.

**Figure 4:**
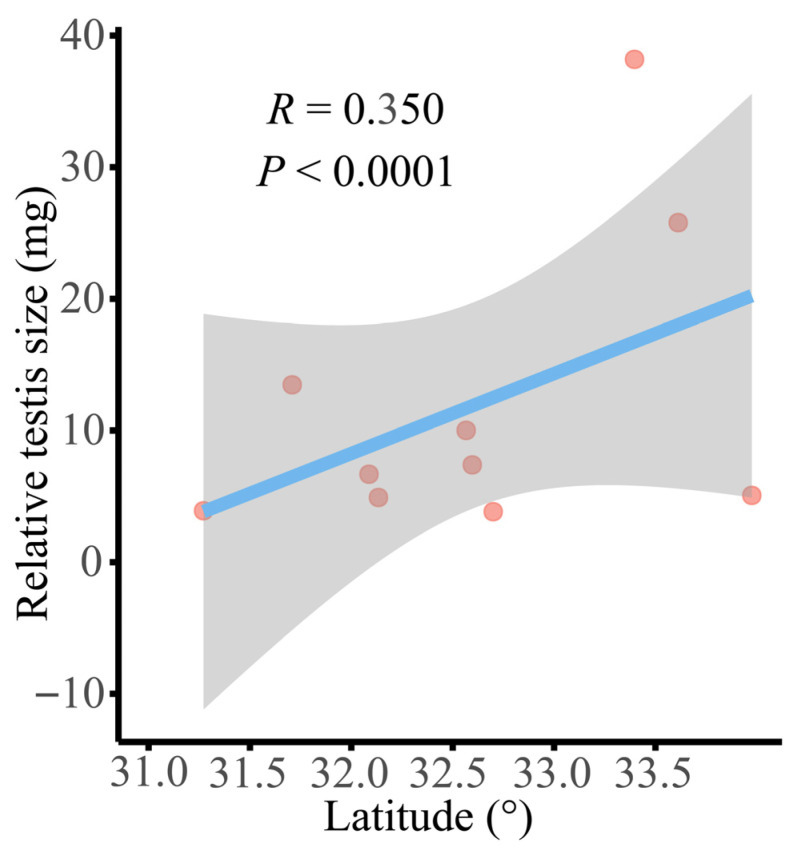
Relationship between the relative testis size in 10 populations of *N. quadranus* and latitude. The blue solid line indicates a significant result of linear regression, and the gray shaded area represents the 95% confidence interval. The red filled circles indicate the mean values of each population.

One-way ANOVA revealed significant interpopulation variation in relative testis size for *N. quadranus* (F_9, 123_ = 6.752, *p* < 0.001; Table S4), with the Wangcang, Wanyuan, and Nanjiang populations exhibiting significantly larger relative testis sizes than other populations. Even after controlling for the effects of age and SVL, the differences in relative testis size among various populations of *N. quadranus* remained significant (ANCOVA: population: F_9, 121_ = 6.610, *p* < 0.001; SVL: F_1, 121_ = 0.352, *p* = 0.554; age: F_1, 121_ = 0.393, *p* = 0.532), indicating that population identity is an independent driver of variation in relative testis size. Additionally, body mass (F_9, 123_ = 5.38, *p* < 0.001), SVL (F_9, 123_ = 3.14, *p* = 0.002) and age (F_9, 123_ = 5.54, *p* < 0.001) were all positively correlated with relative testis size (*p* < 0.05; Figure 3; Table S4). Further regression analysis showed a significant positive correlation between latitude and relative testis size (*r* = 0.350, *p* < 0.001; Figure 4).

### 3.3. Geographical Patterns in Sperm Morphology and Count

**Figure 5:**
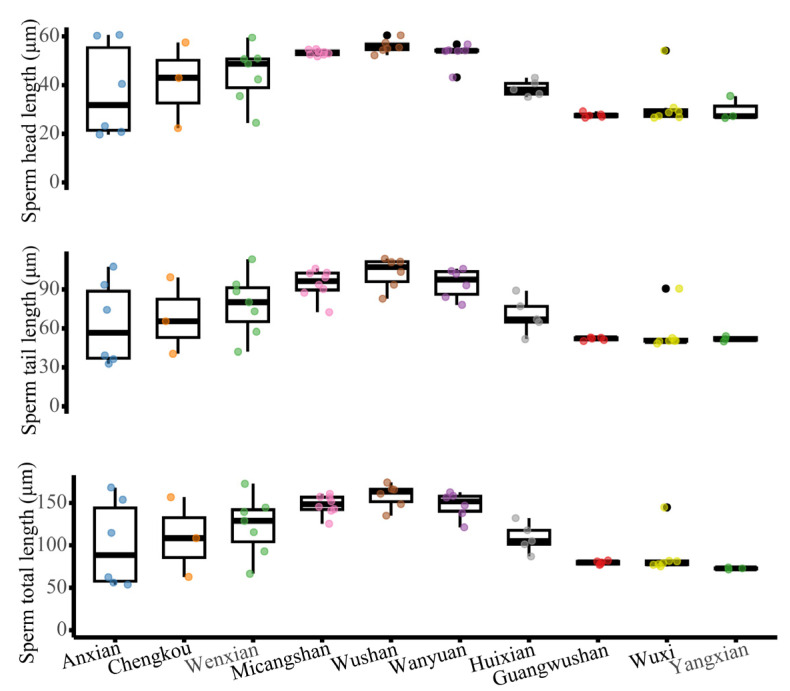
Comparative analysis of sperm dimensions in *N. quadranus* populations. Box plots depict variations in head length, tail length, and total sperm length. The Guangwushan population exhibits concentrated distributions without outliers; Anxian shows greater dispersion, while Wanyuan and Wuxi present distinct outliers.

**Figure 6:**
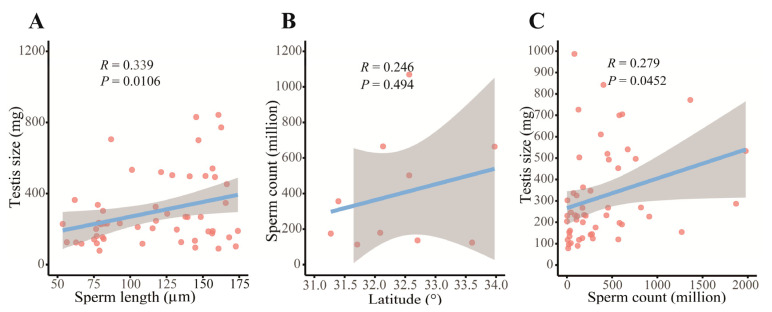
Relationships between testis and sperm traits in *N. quadranus*. (**A**) Association between testis size and sperm length. (**B**) Weak association between sperm count and latitude. (**C**) Association between testis size and sperm count. The blue lines depict the results of the linear regression, the gray shaded regions represent the 95% confidence intervals, and the red dots correspond to the individual data points.

The study found that sperm head and tail length data were obtained from 10 populations. The average sperm head length ranged from 27.67 to 55.8 µm, the average tail length from 51.1 to 102.61 µm, and the overall sperm size from 79.57 to 158.42 µm (Table S5, Figure 5). No significant associations were observed between sperm size and SVL, body mass, or age. Kruskal–Wallis tests revealed significant altitudinal differences in sperm morphology (*p* < 0.001). After controlling for SVL, age, and testis size, significant differences in sperm size among populations were observed (ANCOVA: F_9, 43_ = 3.972, *p* = 0.001). A significant positive correlation existed between sperm length and testis size (GLM: F_1, 54_ = 7.017, *p* = 0.011; Spearman’s: *r* = 0.339, *p* = 0.0106; Figure 6A).

Regarding sperm quantity, significant differences were found among populations (one-way ANOVA: F_9, 54_ = 4.829, *p* < 0.001). After accounting for the effects of body length, age, and testis size, no significant differences in sperm quantity were detected among populations (ANCOVA: F_9, 51_ = 1.508, *p* = 0.170). LSD multiple comparisons showed that the sperm count of the Wuxi population was significantly lower than those of the Wangcang, Wanyuan, and Huixian populations (*p* < 0.05). The Wangcang population had a significantly higher sperm quantity than the Wenxian and Chengkou populations (*p* < 0.05). The Wenxian population had a significantly lower sperm count than the Wanyuan, Huixian, and Nanjiang populations (*p* < 0.05). The Wanyuan population had a significantly higher sperm quantity than the Chengkou population (*p* < 0.05). Overall, sperm count showed a weak increasing trend with latitude (Figure 6B), though this trend was not statistically significant. Additionally, there was a positive correlation between sperm count and testis size (ANOVA: F_1, 62_ = 32.071, *p* < 0.001; Spearman’s: *r* = 0.279, *p* < 0.05; Figure 6C), while no significant correlation was found between sperm count and sperm size (Spearman’s: *r* = 0.252, *p* > 0.05).

### 3.4. Altitudinal Variation in Female Fecundity

**Figure 7:**
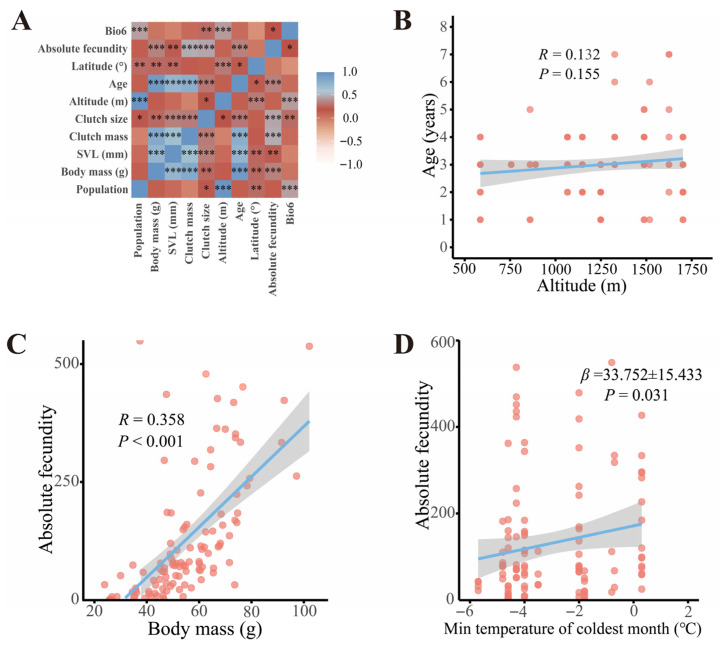
Heatmap and scatter plots of the correlation between geographic and environmental factors and reproductive fecundity-related indices of female *N. quadranus*. (**A**) Heatmap of the correlation between female reproductive fitness and environmental factors. The color gradient represents the correlation coefficient: red indicates a positive correlation (two variables tend to change in the same direction) and blue indicates a negative correlation (two variables tend to change in opposite directions). Significance markers are defined as follows: * denotes significance at the 0.05 level, ** at the 0.01 level, and *** at the 0.001 level. (**B**) Relationship between age and altitude. (**C**) Body mass exhibits a highly significant positive correlation with absolute fecundity. (**D**) The minimum temperature of the coldest month shows a significant positive correlation with absolute fecundity yet only explains 10% of the variation in absolute fecundity, indicating relatively low explanatory power.

One-way ANOVA revealed significant differences in SVL, body mass, age, and clutch mass among populations (Table S6). Heatmap visualizations confirmed weak correlations between SVL and altitude/latitude, consistent with these findings (Figure 7A). Subsequent analysis showed that age increased with altitude (Figure 7B), but after controlling for age, no significant differences in SVL were observed across altitudes or latitudes. Even after controlling for age, population-level differences in female body mass remained nonsignificant (ANOVA: F_11, 105_ = 1.221, *p* = 0.282). GLM analysis identified a significant interaction effect between altitude and age on body mass (F_17, 114_ = 62.158, *p* < 0.001), with age being the primary contributing factor (F_6, 114_ = 122.068, *p* < 0.001). Correlation analysis indicated a strong positive association between body mass and age (Pearson’s *r* = 0.938, *p* < 0.001) and a weak positive correlation with altitude (Pearson’s *r* = 0.12, *p* = 0.17). Heatmap visualization supported these findings by showing positive relationships between body mass and both altitude and age (Figure 7A).

Kruskal–Wallis tests detected significant population differences in absolute fecundity (H_11, 132_ = 45.607, *p* < 0.001). Pairwise comparisons revealed differences in absolute fertility between the Wushan population and Wuxi (*p* = 0.000), Wanyuan (*p* = 0.000), Anxian (*p* = 0.000), Huixian (*p* = 0.000), Nanjiang (*p* = 0.006), Lueyang (*p* = 0.003), and Chengkou (*p* = 0.005) populations. After controlling for SVL, body mass, and age, no significant population differences in absolute fertility remained (ANCOVA: *p* > 0.05), though SVL, body mass, and age all showed significant effects on absolute fertility (ANOVA: SVL: F_2, 129_ = 5.204, *p* < 0.001; body mass: F_2, 129_ = 6.063, *p* < 0.001; age: F_2, 129_ = 6.335, *p* < 0.05). Heatmaps corroborated these findings with strong positive correlations between absolute fertility and SVL, body mass, and age (Figure 7A). Regression analysis further confirmed a significant positive relationship between body mass and absolute fertility after accounting for SVL and age (Spearman’s *r* = 0.664, *p* < 0.001; Figure 7C).

### 3.5. Multivariate Environmental Predictors of Reproductive Traits

Environmental factors influencing male reproductive investment: A total of 31 models were developed with relative testis size as the dependent variable. The best-fit model indicated that Bio2, Bio13, and aspect jointly explained 34.7% of the variation in relative testis size (Table S7). The results revealed a significant negative correlation between relative testis size and the monthly mean of Bio2 (*β* = −0.563 ± 0.098, *p* < 0.001). Furthermore, 63 models were constructed with sperm size as the dependent variable. The best-fit model suggested that slope, aspect, and SEA collectively explained 31.5% of the variation in sperm size (Table S6). The analysis showed a significant positive correlation between sperm size and both aspect (*β* = 0.001 ± 0, *p* < 0.001) and SEA (*β* = 0.006 ± 0.003, *p* < 0.05).

Environmental factors affecting female reproductive investment: The model with the lowest AICc value was selected as the best-fit model. The results indicated that, among the 63 models constructed, female absolute reproductive output was primarily influenced by temperature and topography (Table S8). Specifically, absolute reproductive output exhibited a significant positive correlation with Bio6 (*β* = 33.752 ± 15.433, *p* < 0.05; Figure 7D), though the best-fit model accounted for only 10% of the observed variation in this trait. Given this limited explanatory power, it is likely that additional unmeasured environmental variables contribute to the regulation of female fecundity. These may include factors such as food resource availability, intraspecific competition for breeding resources, or interspecific ecological interactions, as well as potential interactive effects among multiple environmental drivers. Further investigations integrating these variables could help unravel the more complex regulatory mechanisms underlying variation in female reproductive output.

## 4. Discussion

Geographic variation in animal reproductive strategies constitutes a fundamental paradigm for deciphering environmental adaptations. Within vertebrates, established conceptual frameworks integrate principles of energy allocation trade-offs and sexual selection [94,95]. Amphibians, recognized for their heightened sensitivity to environmental perturbations [96], exhibit marked geographic divergence in reproductive strategies; however, the precise patterns and underlying mechanistic drivers remain incompletely understood. Distinct from endotherms that utilize thermoregulation to physiologically buffer reproductive investment, amphibian reproductive strategies appear more susceptible to direct modulation by proximate environmental gradients, including thermal regimes and resource availability. Consequently, this study leveraged *N. quadranus* to elucidate geographically multidimensional adaptations inherent to its reproductive strategy, specifically probing altitudinal clines in energy allocation and dissecting sex-specific responses to the covariation between temperature and precipitation regimes.

### 4.1. Geographic Variation in Male Reproductive Investment: Testicular and Sperm Traits

Geographic variation in male reproductive traits was evident in *N. quadranus*, particularly in relative testis size. A significant positive correlation was detected between relative testis size and latitude (*p* < 0.05), suggesting that individuals from higher-latitude populations allocate more reproductive energy to testicular development. This finding aligns with life-history theory, which posits that increased sexual selection pressure drives greater investment in reproductive organs. This pressure is often associated with shorter breeding seasons and intensified sperm competition at higher latitudes. Similar patterns have been reported in *Fejervarya limnocharis* and *Polypedates megacephalus* [97,98]. However, testis size negatively correlates with altitude in species like *Rana temporaria* and *Hyla gongshanensis jingdongensis* [29,31]. These differences demonstrate that geographic effects on testis size are species-specific, shaped by local ecological pressures (e.g., habitat heterogeneity, pollutant exposure), mating system variation (e.g., polygamy intensity), and evolutionary history, including parental care strategies [99,100,101].

Notably, the role of age in testicular development exhibited a complex pattern. While relative testis size showed a positive correlation with age, this effect disappeared after controlling for SVL, and significant interpopulation differences persisted. Thus, although age covaries with relative testis size, geographic divergence constitutes the primary determinant. This likely reflects stronger population-level variation in sexual selection pressures (e.g., sperm competition intensity) compared to within-population ontogenetic effects.

No significant differences in testicular asymmetry were detected, indicating bilateral symmetry as a stable reproductive strategy in this species [60,71,102], aligning with a sperm competition-dominated reproductive system. This pattern contrasts with the classical compensatory hypothesis [71], which suggests unilateral testicular enlargement as a mechanism to offset reduced function in the contralateral testis. Moreover, testicular symmetry showed no correlation with age, suggesting stability across ontogeny and minimal senescence-related effects. Notably, relative testis size in *N. quadranus* ranks above 90% of more than 130 surveyed amphibian species [103], reinforcing the importance of sperm competition in shaping reproductive strategy. An exception was observed in the high-altitude population (1518 m), which exhibited significantly greater asymmetry than lower-elevation populations. This deviation, confirmed by re-analysis of raw data, is unlikely due to measurement error. Two explanations are plausible: (i) developmental plasticity induced by microenvironmental stressors (e.g., extreme cold or resource scarcity during critical developmental windows) [104], or (ii) stochastic developmental noise, which produces individual asymmetry without altering population-wide symmetry patterns [105]. This process can generate individual-level asymmetry. However, it typically maintains population-wide symmetry patterns. Future studies should expand altitudinal sampling and integrate testicular morphometrics with sperm traits to assess whether this asymmetry reflects adaptive plasticity or neutral variation.

Our study revealed significant intrapopulation variation in testis size among *N. quadranus* populations, which showed no correlation with body size. Sperm count exhibited an increasing trend with latitude, indicating that high-latitude populations tend to adopt a reproductive strategy prioritizing quantity over quality. This pattern may reflect an adaptive response to increased post-copulatory sexual selection pressure: under more intense sperm competition, producing larger sperm counts could enhance fertilization success [102]. The observed covariation between testis size and sperm number supports the central tenet of sperm competition theory [106,107,108]. Additionally, sperm length also exhibited a positive association with testis size, lending support to the long-sperm advantage hypothesis, which proposes that larger sperm confer enhanced competitive performance [109]. This study found no significant correlation between sperm size and age, suggesting that sperm traits are relatively stable at the population level, shaped more by genetic and environmental factors than by ontogeny. Furthermore, population-level differences in sperm count became non-significant after controlling for age, body size, and testis size, suggesting that age may indirectly affect sperm number through its effects on testicular development.

Taken together, the concurrent increase in sperm count and length suggests that *N. quadranus* may simultaneously invest in both aspects of ejaculate quality to optimize reproductive success across environmental gradients. Collectively, these results illuminate how spatial heterogeneity shapes reproductive tactics and underscore the necessity of environmental integration in amphibian life-history analyses.

### 4.2. Geographic Patterns in Female Fecundity and Life-History Traits

The results of this study indicate that female *N. quadranus* exhibit a body mass-dependent reproductive strategy. A strong positive correlation was found between absolute fecundity and body mass, whereas the influence of SVL was limited, and age had no significant effect after controlling for covariates. Body mass serves as a central integrator of the pace-of-life continuum in amphibians, shaping multidimensional traits including growth rates, timing of sexual maturity, and reproductive allocation strategies [44]. Although age did not directly affect fecundity, it exerted a strong indirect influence: female body mass increased significantly with age, suggesting that older individuals accumulate greater somatic reserves through prolonged resource acquisition, thereby enhancing reproductive output. These findings align with patterns documented in montane amphibians, such as *Hyla labialis* and *Rana temporaria* [110,111], indicating that body mass serves as a robust proxy for energy reserves governing reproductive investment. Age thus acts primarily as a temporal scaffold for energy accumulation, indirectly shaping reproductive capacity.

From a physiological perspective, increased body mass may enlarge the coelomic cavity, thereby alleviating spatial constraints on clutch size. Additionally, greater somatic energy reserves facilitate oogenesis and provide necessary metabolic support during reproduction. Given that *N. quadranus* follows a polyandrous mating system, females may selectively allocate reproductive resources to maximize reproductive success under sperm competition and environmental uncertainty. Age was positively correlated with altitude, indicating that females in high-altitude populations tend to be older, reflecting an adaptive strategy of delayed sexual maturity. This “slow growth-high reserves” life-history strategy, documented in other high-altitude amphibians such as *Bufo minshanicus* [112], allows females to reproduce successfully in environments characterized by low temperatures, short growing seasons, and scarce resources.

Populations from higher altitudes and latitudes exhibited larger average female body mass and greater absolute fecundity, indicating elevated reproductive investment in response to environmental constraints. This trend is consistent with patterns observed in *Hyla labialis*, *Bufo andrewsi* [76,111], and other amphibians inhabiting montane regions. High-altitude environments are typically characterized by lower temperatures, shorter breeding seasons, and limited food availability. To offset these constraints, females often produce fewer but larger eggs per reproductive event, enhancing offspring survival and fitness [19,113]. Conversely, populations inhabiting lower elevations with stable, extended breeding seasons preferentially employ high-fecundity strategies featuring smaller eggs, thereby maximizing reproductive output through successive breeding events. Importantly, once age was controlled for, population-level differences in body mass were no longer significant, while fecundity variation remained closely tied to body mass. This indicates that geographic variation in female reproduction is driven primarily by divergent energy allocation strategies rather than age structure per se, with age serving as a mechanism that indirectly shapes body mass through extended growth. Additionally, phylogenetic history, ecological niche specialization, and species-specific life-history constraints collectively shape female reproductive strategies [49,114]. Therefore, future comparative studies should incorporate these multidimensional factors to fully elucidate the adaptive mechanisms underlying fecundity variation in amphibians.

### 4.3. Environmental Determinants of Reproductive Strategy Divergence

This study reveals that temperature, precipitation, and topography significantly regulate reproductive trait differentiation in *N. quadranus*. In males, relative testis size was primarily influenced by Bio2, NDVI, and aspect. These factors together suggest that stable low-temperature environments may facilitate enhanced testicular development. Sperm length showed a strong positive association with both topographic complexity and SEA, collectively explaining 35% of the observed variation. Topography acts as both a physical constraint and a selective force, influencing reproductive adaptation through its impact on aquatic breeding habitats. Variations in slope, elevation, and microtopography directly influence critical aquatic and semi-aquatic microhabitats for reproduction, creating hydrologically diverse environments (e.g., torrential streams and stagnant pools). In fast-flowing streams, elongated flagella enhance motility against currents, whereas in still waters, larger sperm heads may facilitate egg membrane penetration [115]. Additionally, complex topography correlates with resource heterogeneity, providing richer microclimates and greater prey availability that enable increased energy allocation toward costly sperm production [83]. By contrast, SEA-induced moisture instability indirectly regulates habitat quality by amplifying environmental fluctuations, intensifying sperm competition pressures. In regions characterized by significant SEA, males require higher-quality sperm to adapt to unstable aquatic conditions. Ultimately, topography functions as both a microhabitat constraint and adaptive driver, reinforcing its association with sperm elongation. This suggests that elongated sperm in *N. quadranus* represent an adaptive response to ecological constraints in energetically demanding or unstable habitats [84].

Notably, *N. quadranus* exhibits no evolutionary trade-off between sperm length and sperm count. Male reproductive investment is primarily driven by rainfall seasonality and the duration of the breeding season, the latter being constrained by resource availability. Environmental heterogeneity further enhanced this regulatory flexibility. In stable environments, rainfall dominated as the key driver, with sperm count serving as the primary reproductive tactic. In challenging habitats, sperm length regulation was predominantly influenced by energy allocation. Resource limitation in low-rainfall regions intensified male competition and elevated reproductive investment—consistent with convergent adaptation patterns observed in *Duttaphrynus melanostictus* [116]. Cross-species comparisons further support environmentally driven convergence: phenology in the *Eurycea bislineata* species complex negatively correlated with mean annual temperature [117], while sperm count in *Leptodactylus podicipinus* was strongly associated with precipitation [78]. These parallels support a broader framework of environment-driven reproductive convergence across amphibians.

In female *N. quadranus*, variations in absolute fecundity are significantly influenced by Bio6 and aspect, which together account for 10% of the observed variation. Environmental variables also shape female reproductive investment; however, their individual explanatory power proves limited. For instance, the best-fit model indicates that Bio6 alone explains only 10% of the variation in absolute fecundity. This suggests that fecundity is modulated by the synergistic effects of multiple environmental factors, including resource availability, intraspecific and interspecific competition, and climate–topography interactions extending beyond simple thermal constraints. The decline in reproductive output under reduced winter temperatures further underscores temperature’s restrictive role in reproductive physiology.

### 4.4. Conservation Implications

Geographic variation in *N. quadranus*’s reproductive strategies, correlated with altitude, latitude, and temperature, demonstrates adaptive responses to environmental heterogeneity. These patterns demonstrate that reproductive investment is not uniform but context-dependent, with males primarily regulated by diurnal temperature range and precipitation, while females are constrained by body mass and winter thermal conditions. Such sex-specific strategies reveal the multidimensional nature of adaptation and underscore that conservation efforts must account for ecological and demographic heterogeneity among populations.

Given the species’ current IUCN Near Threatened status, conservation planning should prioritize habitat-specific management. Populations at high altitudes and latitudes, which exhibit delayed maturation and larger female body mass, require sufficient breeding refugia and stable thermal microhabitats to support successful reproduction. Conversely, low-altitude populations with higher fecundity but smaller eggs show greater dependence on continuous aquatic habitats and stable hydrological regimes. Protecting habitat mosaics encompassing this ecological diversity is therefore essential for maintaining overall population resilience.

Furthermore, the strong correlations between reproductive traits and climatic variables such as minimum winter temperature and rainfall seasonality indicate that *N. quadranus* is particularly vulnerable to climate change. Rising temperature extremes and altered precipitation patterns may disrupt breeding synchrony, reduce sperm count, or constrain female energy accumulation, ultimately threatening reproductive success. Integrating long-term monitoring of reproductive traits with climate projections will thus provide early-warning indicators of population decline.

## 5. Conclusions

This study elucidates the adaptive mechanisms of reproductive strategies in *N. quadranus* in response to geographic variation. Male relative testis size increased significantly with latitude, while sperm count showed a weak positive association with latitude that was not statistically significant. Meanwhile, sperm length is positively correlated with topographic complexity and evapotranspiration variability. These findings highlight sperm competition as a major evolutionary force in shaping male reproductive traits. Female absolute fecundity is positively associated with body mass, which in turn is influenced by altitude and min winter temperature. Reproductive investment in both sexes is modulated by key bioclimatic variables—mean diurnal range (Bio2), minimum temperature of the coldest month (Bio6), and precipitation of the wettest month (Bio13)—underscoring the role of environmental heterogeneity in driving life-history trait divergence across populations. Collectively, this work establishes a framework for understanding amphibian reproductive adaptations across environmental gradients. However, the limitations of this study include the failure to assess the possible trade-off between ovary size and egg size, as well as the lack of research on aspects such as behavior, resource allocation, and mating success. Additionally, the molecular mechanisms underlying the plasticity of reproductive traits remain unresolved. To further deepen the understanding, future research should combine genomic tools with environmental monitoring to explore the molecular basis and ecological consequences of reproductive plasticity in amphibians under the context of climate change.

## Data Availability

The data presented in this study are available on request from the corresponding author.

## Supplementary Materials

The following supporting information can be downloaded at https://www.mdpi.com/article/10.3390/biology14091224/s1: Table S1: Study site locations and brief descriptions. Table S2: The information on 25 environmental variables. All data below were accessed on 15 September 2022. Table S3: Measured values of left and right testis mass (mg) and total testis mass (mg) for *Nanorana quadranus* populations sampled from ten localities in China. Values in descending order are mean, SD and range. Table S4: Comparison of SVL, age and relative testis size among male populations of *N. quadranus* (mean ± standard deviation). Table S5: Measured values of sperm head, tail and total length (μm) for *N. quadranus* populations sampled from 10 localities in China. Mean ± SD range. Table S6: Comparisons of reproductive life history traits of *N. quadranus* from twelve altitudes in China. The statistical description value is mean ± SD. Table S7: Multiple regression models of male relative testis size, sperm length and environmental predictors in *N. quadranus*. Table S8: A multiple regression model of absolute fecundity and environmental predictors in *N. quadranus*.
