# Supplementary material for: Reproductive Strategies of the Swelled Vent Frog (Nanorana quadranus): Testicular Size, Sperm Traits, and Fecundity Responses to Geographical Gradients
Source: Biology (Basel). 2025 Sep 9;14(9):1224. doi: 10.3390/biology14091224 (PMC12467736; doi:10.3390/biology14091224)
Supplement: Supplementary file 1 [file biology-14-01224-s001.zip › biology-3785484-supplementary.pdf]

Reproductive Strategies of the Swelled Vent Frog (*Nanorana quadranus*): Testicular Size, Sperm Traits, and Fecundity Responses to Geographical Gradients

Lulu Lyu [1], Shuang Huang [1], Miao He [1] and Yan Huang [1,2*]

1 Key Laboratory of Southwest China Wildlife Resources Conservation (Ministry of Education), China West Normal University, Nanchong 637009, China; 15881710835@163.com (L.L.); shuang_huang1230@163.com (S.H.); 17609268594@163.com (M.H.)

2 Key Laboratory of Artificial Propagation and Utilization in Anurans of Nanchong City, China West Normal University, Nanchong 637009, China

* Correspondence: sunflower-hy@126.com

Table S1. Study site locations and brief descriptions

Number	Population	Situation	Latitude and longitude	Elevation	Number of males	Number of females	Total
1	Wuxi	Xujia Town, Wuxi County, Banan District, Chongqing municipality	109.60069; 31.27111	586	17	16	33
2	Wangcang	Moon Township, Wangcang County, Guangyuan City, Sichuan Province	106.55908; 32.59732	756	9	3	12
3	Wenxian	Wenxian County, Longnan City, Gansu Province	105.09679;	860	10	5	15

			32.70333				
4	Foping	Foping County, Hanzhong City, Shaanxi Province	108.00672; 33.51361	890	0	3	3
5	Wanyuan	HuMountain National Nature Reserve, Yuancheng City, Dazhou City, Sichuan Province	108.11598; 32.13377	1066	21	8	29
6	Yangxian	Yanfeng Village, Yang County, Hanzhong City, Shaanxi Province	107.41203; 33.60417	1148	8	18	26
7	Wushan	Chongqing Banan District, Wushan County, Qingshan Residence	110.01034; 31.39667	1249	15	18	33
8	Anxian	Qianfo Town, An District, Mianyang City, Sichuan Province	104.25767; 31.70826	1326	22	11	33
9	Fengjie	Xinglong Town, Fengjie County, Banan District, Chongqing municipality	109.28237; 30.38341	1480	2	4	6
10	Huixian	Houjiagou, Hui County, Longnan City, Gansu Province	105.86727; 33.97528	1488	10	19	29
11	Nanjiang	Sichuan Bazhong, Nanjiang, County Guangwu Mountain 18 moon pool	106.69014; 32.56585	1518	14	9	23

12	Lueyang	Shaanxi Hanzhong City, Luoyang, County Wulong Cave National Park	106.28531; 33.59056	1625	0	8	8
13	Chengkou	Fangdouping, Chengkou County, Banan District, Chongqing municipality	108.61244; 32.08716	1702	7	18	25

Note: The data in the table represent the total sampling data, while the sample size mentioned in the paper refers to the number of samples after excluding measurement errors.

Table S2. The information on 25 environmental variables. Accessed on 15 September 2022 for all Webs below.

ID	Abbreviation	Name	Data Source
1	Bio1	Annual Mean Temperature	https://www.worldclim.org/
2	Bio2	Mean Diurnal Range	https://www.worldclim.org/
3	Bio3	Isothermality	https://www.worldclim.org/
4	Bio4	Temperature Seasonality	https://www.worldclim.org/
5	Bio5	Maximum Temperature of Warmest Month	https://www.worldclim.org/
6	Bio6	Minimum Temperature of Coldest Month	https://www.worldclim.org/
7	Bio7	Temperature Annual Range	https://www.worldclim.org/
8	Bio8	Mean Temperature of Wettest Quarter	https://www.worldclim.org/
9	Bio9	Mean Temperature of Driest Quarter	https://www.worldclim.org/
10	Bio10	Mean Temperature of Warmest Quarter	https://www.worldclim.org/
11	Bio11	Mean Temperature of Coldest Quarter	https://www.worldclim.org/
12	Bio12	Annual Precipitation	https://www.worldclim.org/
13	Bio13	Precipitation of Wettest Month	https://www.worldclim.org/

14	Bio14	Precipitation of Driest Month	https://www.worldclim.org/
15	Bio15	Precipitation Seasonality	https://www.worldclim.org/
16	Bio16	Precipitation of Wettest Quarter	https://www.worldclim.org/
17	Bio17	Precipitation of Driest Quarter	https://www.worldclim.org/
18	Bio18	Precipitation of Warmest Quarter	https://www.worldclim.org/
19	Bio19	Precipitation of Coldest Quarter	https://www.worldclim.org/
20	PET	Potential Evapotranspiration	https://www.usgs.gov/products/data
21	AET	Actual Evapotranspiration	https://www.usgs.gov/products/data
22	SEA	Seasonal ET Anomaly	https://www.usgs.gov/products/data
23	NDVI	Normalized Difference Vegetation Index	https://www.resdc.cn/
24	Slope	Slope	http://www.gscloud.cn/
25	Aspect	Aspect	http://www.gscloud.cn/

Table S3. Measured values of left and right testis mass (mg) and total testis mass (mg) for *N. quadranus* populations sampled from ten localities in China. Values in descending order are mean, SD and range

Number	Elevation	Population	Measured values	Left testis mass (mg)	Right testis mass (mg)	Total testis mass (mg)	*t*	*p*
1	586	Wuxi *n* = 17	Mean ± SD; Range	101.64 ± 64.97; 0.239 - 0.0130	96.32 ± 60.26; 225.7 - 13.3	197.95 ± 124.54; 464.8 - 26.3	1.58	0.13
2	756	Wangcang *n* = 9	Mean ± SD; Range	366.42 ± 178.99; 643.6-158	328.27 ± 190.26; 641.4 - 84.4	694.69 ± 361.44; 1245.4 - 286.8	1.5	0.17
3	860	Wenxian *n* = 10	Mean ± SD; Range	111.57 ± 68.14; 271.5-53.7	106.4 ± 60.8; 231.9 - 49	217.97±128.07; 503.4-102.7	0.98	0.35
4	1066	Wanyuan *n* = 21	Mean ± SD; Range	719.41 ± 786.76; 2878.9-48	684.64 ± 727.83; 2709 - 48.7	1404.05 ± 1512.6; 5587.9 - 96.7	9	0.35
5	1148	Yangxian *n* = 8	Mean ± SD; Range	159.21 ± 63.94; 300-101.4	170.39 ± 68.66; 300 - 98.9	329.6 ± 131.51; 600 - 200.3	1.64	0.12
6	1249	Wushan *n* = 15	Mean ± SD; Range	129.87 ± 85.31; 299.5-23.2	129.09 ± 87.48; 281.3-20.8	258.95 ± 171.9; 580.8 - 44	1.79	0.12
7	1326	Anxian *n* = 22	Mean ± SD; Range	108.31 ± 50.73; 272.2 - 34.9	103.1 ± 57.52; 318 - 43.1	211.41 ± 107.5; 590.2 - 78	0.17	0.87

8	1488	Huixian n = 10	Mean ± SD; Range	175.34 ± 98.89; 347.8 - 67.5	178.97 ± 89.38; 357.3 - 65.8	354.31 ± 186.61; 705.1 - 133.3	1.71	0.1
9	1518	Nanjiang n = 14	Mean ± SD; Range	879.04 ± 950.7; 2835.1 - 71.3	952.22 ± 1063.16; 3458.9-80.8	1831.26 ± 2008.49; 6294 - 154.5	-0.43	0.68
10	1702	Chengkou n = 7	Mean ± SD; Range	113.1 ± 120.03; 381.2 - 44.6	107.96 ± 95.98; 320.2-36.4	221.06 ± 215.81; 701.4 - 81	-1.48	0.16

Table S4. Comparison of SVL, Age and relative testis size among male populations of *N. quadranus* (Mean ± standard deviation)

Number	Elevation	Population	Latitude and longitude	Body mass (g)	SVL (mm)	Age (years)	Testis mass (mg)	Relative testis size (mg)
1	586	Wuxi *n* = 17	109.6006; 31.27111	47.6 ± 15.17	69.92 ± 6.93	1.88 ± 0.93	197.95 ± 124.54	3.9 ± 1.6
2	756	Wangcang *n* = 9	106.5594; 32.59649	54.53 ± 18.71	74.49 ± 9.39	2.89 ± 1.36	694.69 ± 361.44	7.39 ± 4.32
3	860	Wenxian *n* = 10	105.0996; 32.69972	43.55 ± 9.56	66.84 ± 8.91	1.9 ± 0.74	217.97 ± 128.07	3.83 ± 1.64
4	1066	Wanyuan *n* = 21	108.116; 32.13376	49.86 ± 10.87	72.87 ± 4.99	2.33 ± 0.86	1351.45 ± 1496.66	4.91 ± 3.37
5	1148	Yangxian *n* = 8	107.4123; 33.61222	37.06 ± 11	72.58 ± 5.00	1.75 ± 0.71	329.60 ± 131.51	25.79 ± 24.74
6	1249	Wushan *n* = 15	110.0103; 31.39667	33.73 ± 5.83	68.69 ± 3.96	1.20 ± 0.41	258.95 ± 171.90	38.19 ± 37.5
7	1326	Anxian *n* = 22	104.2577; 31.70829	54.6 ± 10.8	77.52 ± 5.31	2.73 ± 0.88	211.41 ± 107.51	13.47 ± 7.4
8	1488	Huixian *n* = 10	105.8672; 33.97528	53.38 ± 8.66	77.66 ± 4.9	2.6 ± 0.52	354.31 ± 186.61	5.07 ± 2.94
9	1518	Nanjiang *n* = 14	106.6902; 32.56639	45.59 ± 9.55	72.16 ± 5.1	2.14 ± 0.53	1816.81 ± 1936.24	10.01 ± 6.57
10	1702	Chengkou *n* = 7	108.6124; 32.08712	40.95 ± 8.82	67.14 ± 8.52	1.55 ± 0.69	158.66 ± 191.05	6.68 ± 3.53

Table S5. Measured values of sperm head, tail and total length (µm) for *N. quadranus* populations sampled from 10 localities in China. Mean ± SD range

Number	Elevation	Population	Measured values	Sperm head length (µm)	Sperm tail length (µm)	Total sperm length (µm)
1	586	Wuxi *n* = 6	Mean ± SD; Range	31.94 ± 9.89; 54.13 - 26.64	26.64 ± 54.13; 90.63 - 48.31	88.08 ± 25.10; 144.76 - 75.15
2	756	Wangcang *n* = 3	Mean ± SD; Range	53.31 ± 1.06; 54.81 - 51.78	94.28 ± 10.98; 106.08 - 72.45	147.6 ± 11.51; 160.68 - 125.43
3	860	Wenxian *n* = 5	Mean ± SD; Range	27.67 ± 1.02; 29.25 - 26.65	51.9 ± 1.16; 53.02 - 50.31	79.57 ± 2.08; 82.07 - 76.95
4	1066	Wanyuan *n* = 5	Mean ± SD; Range	52.7 ± 4.79; 56.67 - 43.15	94.56 ± 11.51; 105.93 - 78.08	147.25 ± 15.52; 162.6 - 121.24
5	1148	Yangxian *n* = 8	Mean ± SD; Range	28.68 ± 1.8; 30.75 - 27.44	51.1 ± 2.33; 53.34 - 48.69	79.78 ± 3.89; 84.09 - 76.53
6	1249	Wushan *n* = 6	Mean ± SD; Range	55.8 ± 2.8; 60.44 - 52.24	102.61 ± 12.19; 113.63 - 82.81	158.42 ± 14.22; 174.07 - 135.06
7	1326	Anxian *n* = 7	Mean ± SD; Range	37.65 ± 19.32; 60.6 - 19.83	64.2 ± 32.48; 107.48 -	101.86 ± 51.63;

					32.72	168.08 - 53.55
8	1488	Huixian *n* = 7	Mean ±SD; Range	38.67 ± 3.22; 42.99 - 35.09	69.93 ± 13.97; 89.07 - 51.75	108.6 ± 17.13; 132.06 - 86.84
9	1518	Nanjiang *n* = 6	Mean ± SD; Range	27.67 ± 1.02; 29.25 - 26.65	51.9 ± 1.16; 53.02 - 50.31	79.57 ± 2.08; 82.07 - 76.95
10	1702	Chengkou *n* = 3	Mean ± SD; Range	40.89 ± 17.67; 57.47 - 22.3	68.42 ± 29.52; 99.29 - 40.46	109.31 ± 47.01; 156.77 - 62.76

Table S6. Comparisons of reproductive life history traits of *N. quadranus* from twelvealtitudes in China. The statistical description value is Mean ± SD.

Number	Population、Sample size	Body mass (g)	SVL (mm)	Age (years)	Weight of ovary	Absolute fertility
1	Wuxi *n* = 16	54.07 ± 4.29	78.74 ± 2.23	2.88 ± 0.27	1.35 ± 0.28	415.35 ± 105.57
2	Wangcang *n* = 3	58.77 ± 3.44	86.71 ± 0.95	3	0.95 ± 0.12	77.31 ± 37.05
3	Wenxian *n* = 5	45.04 ± 6.68	73.89 ± 3.41	2.00± 0.45	0.74 ± 0.14	37.57 ± 10.77
4	Foping *n* = 3	56.7 3± 2.96	85.61 ± 1.71	3	1.13 ± 0.27	237.43 ± 122.68
5	Wanyuan *n* = 18	61.07 ± 3.07	81.83 ± 1.47	3.33 ± 0.24	1.36 ± 0.25	495.26 ± 119.14
6	Yangxian *n* = 14	53.4 ± 2.24	84.05 ± 1.29	2.79 ± 0.19	0.80 ± 0.05	73.83 ± 11.07
7	Wushan *n* = 14	41.66 ± 2.55	72.73 ± 2.97	1.93 ± 0.20	0.46 ± 0.07	20.46 ± 10.97
8	Anxian *n* = 8	66.59 ± 6.02	90.08 ± 2.81	4.25 ± 0.53	1.29 ± 0.20	259.38 ± 75.41
9	Huixian *n* = 19	63.43 ± 4.3	84.51 ± 2.36	3.37 ± 0.24	1.04 ± 0.10	192.4 ± 39.21
10	Nanjiang *n* = 6	53.03 ± 5.4	80.68 ± 3.22	2.50 ± 0.34	1.43 ± 0.36	455.7 ± 168.43
11	Lveyang *n* = 8	77.45 ± 7.81	90.2 ± 3.08	4.63 ± 0.70	1.37 ± 0.16	287.19 ± 72.62
12	Chengkou *n* = 18	51.89 ± 3.22	79.48 ± 1.84	2.61 ± 0.22	0.76 ± 0.08	259.72 ± 99.99
	F	4.180	4.169	5.296	2.678	2.890
	p	0	0	0	0.004	0.002

Note: F = test value of one-way ANOVA for differences in population means. N = sample size. ******p* **<0.001**

Table S7. Multiple regression models of male relative testis size, sperm size and environmental predictors in *N. quadranus*.

Prediction model	R^2	AICc	Delta AICc	AICc wi	*p*
**Relative testis size**					
Best model*	0.347	102.067	0	0.441	< 0.001
Mean diurnal temperature range (Bio2)、(Aspect)	0.287	109.473	7.406	0.011	< 0.001
Mean diurnal temperature range (Bio2)	0.201	122.515	20.448	< 0.001	< 0.001
Normalized Difference Vegetation Index (NDVI)	0.07	142.715	40.648	< 0.001	0.002
Precipitation in the wettest month (Bio13)	0.021	149.496	47.429	< 0.001	0.093
Seasonal anomaly of evapotranspiration (SEA)	0.012	150.716	48.649	< 0.001	0.203
Aspect	0.007	151.436	49.369	< 0.001	0.339
**Sperm size**					
Best model*	0.315	-65.294	0	0.226	0.001
Aspect	0.196	-61.122	4.172	0.028	0.001
Seasonal anomaly of evapotranspiration (SEA)	0.072	-53.078	12.216	0.002	0.045
Normalized Difference Vegetation Index (NDVI)	0.042	-51.285	14.01	< 0.001	0.13
Precipitation in the hottest quarter (Bio18)	0.017	-49.814	15.48	< 0.001	0.345
Mean diurnal temperature range (Bio2)	0.01	-49.433	15.862	< 0.001	0.467

Slope	< 0.001	48.893	16.401	< 0.001	0.911

Note: The best model for relative testis size *includes NDVI , Bio2, Bio13, and aspect; the best model for sperm size *includes aspect, slope, and SEA.

Table S8. A multiple regression model of absolute fecundity and environmental predictors in *N. quadranus*.

Prediction model	R^2	AICc	Delta AICc	AICc wi	*p*
Best model	0.1	1910.306	0	0.175	0.001
Minimum Temperature of Coldest Month (Bio6)	0.035	1917.354	7.048	0.005	0.031
Aspect	0.014	1920.238	9.931	0.001	0.174
The potential evapotranspiration (AET)	0.018	1919.744	9.438	0.002	0.127
Normalized Difference Vegetation Index (NDVI)	0.014	1920.196	9.89	0.001	0.169
The rainfall of the hottest quarter (Bio18)	0.01	1920.836	10.53	< 0.001	0.261
Seasonal Anomaly Evapotranspiration (SEA)	0.001	1921.982	11.676	< 0.001	0.710

Note: The best model included factors such as Bio6 and aspect.
